# Induction of ICAM-1 Expression in Mouse Embryonic Fibroblasts Cultured on Fibroin-Gelatin Scaffolds

**Published:** 2017

**Authors:** M. A. Nosenko, N. V. Maluchenko, M. S. Drutskaya, A. Y. Arkhipova, I. I. Agapov, S. A. Nedospasov, M. M. Moisenovich

**Affiliations:** Faculty of Biology, Lomonosov Moscow State University, Leninskie Gory 1, bldg. 12, Moscow, 119234 , Russia; Laboratory of Molecular Mechanisms of Immunity, Engelhardt Insitute of Molecular Biology, Vavilov Str. 32, Moscow, 119991, Russia; Laboratory of Bionanotechnologies, Shumakov Federal Scientific Center for Transplantology and Artificial Organs, Schukinskaya Str. 1, Moscow, 123182, Russia; German Rheumatism Research Center, Chariteplatz 1, Berlin, 10117, Germany

**Keywords:** MEF, bioengineering, polymeric matrix, stromal cells, ICAM-1, 3D culture

## Abstract

Culturing of allogeneic or autologous cells in three-dimensional bioresorbable scaffolds is an important step in the engineering of constructs for regenerative medicine, as well as for experimental systems to study the mechanisms of cell differentiation and cell-to-cell interaction. Artificial substrates can modulate the phenotype and functional activity of immobilized cells. Investigating these changes is important for understanding the fundamental processes underlying cellular interactions in a 3D microenvironment and for improving tissue-engineered structures. In this study, we investigated the expression of the ICAM-1 adhesion molecule in mouse embryonic fibroblasts (MEF) when cultured on gelatin-fibroin scaffolds. Increased expression of ICAM-1 in MEF was detected only under 3D culture conditions both at the mRNA and protein levels. At the same time, the MEF cultured on various substrates did not oerexpress MAdCAM-1, indicating the selective effect of 3D culture conditions on ICAM-1 expression. One possible mechanism for ICAM-1 induction in MEF is associated with the activation of AP-1, since expression of c-Fos and Junb (but not cJun and Jund) was increased in MEF in 3D. When cultured under 2D conditions, the expression level of AP-1 components did not change.

## INTRODUCTION

We have previously engineered a fibroin-gelatin sponge scaffold which provides a substrate for the adhesion and proliferation of various cell types [1]. Subcutaneous injection of 200–400 μm fragments of this scaffold facilitated the regeneration of deep skin wounds in mice, apparently because of their immunomodulating activity [2]. One possible mechanism underlying the regenerative activity of gelatin-fibroin scaffolds could be an increase in the expression of adhesion molecules, which are involved in immune responses, by fibroblasts after they come into contact with the scaffold surface. Presumably, ICAM-1 is one of such molecules. Normally, only a small amount of ICAM-1 is present on fibroblasts, but changes in the microenvironment can lead to an increase in its expression. In line with this fact, the inflammatory response results in a significant increase in ICAM- 1 expression by tissue-specific fibroblasts, which in turn promotes the migration of the immune cells to the site of the inflammation [3, 4]. Moreover, ICAM-1 is important for the functioning of lymphoid organs, where this molecule facilitates contact interactions between immune, stromal, and endothelial cells [5]. Thus, reconstitution of these and other interactions mediated by the 3D environment both *in vitro *and *in vivo *is an important step in the engineering of artificial lymphoid tissue [6].

## MATERIALS AND METHODS

A primary culture of MEF, as well as sponge fibroin scaffolds supplemented with 30% gelatin (3D FG), was prepared as previously described [1]. For the generation of fibroin-gelatin films, the same aqueous solution as that for 3D scaffolds was used. Firboin-gelatin films or Nunc culture plastic (Thermo Fisher Scientific, USA) were used for a 2D culture.

Total RNA was isolated from MEFs and analyzed according to the standard protocol using the TRI Reagent (Sigma Aldrich, USA), reverse transcription kits (Thermo Scientific, EN0521 and K1621), and a real-time PCR kit (Synthol M-440) in compliance with the manufacturer’s recommendations. The quality of the reactions was evaluated using a melting curve analysis and the electrophoresis of amplification products in 1.8% agarose gel. Photographs of the gels were prepared using the GelDoc^™^; XR+ System (BioRad, USA). Total RNA isolated from murine lymph nodes was used as a positive control in the analysis of *Madcam1 *gene expression. A quantitative PCR analysis was performed on a 7500 RT-PCR System instrument (Applied Biosystems, USA).

Immunofluorescence staining was performed using αICAM1-Cy5 antibodies (KAT1), nuclear dye SYTOX orange, and FITC-phalloidin conjugate to visualize polymerized actin. The samples were embedded in Aqua-Poly/Mount (Polysciences,USA) and examined using an electron microscope Camscan Series II (Cambridge Instruments) in the SEI mode and a Nikon Eclipse Ti-E microscope with a confocal module A1 (Nikon Corp., Japan) and Apo TIRF 60×/1.49 Oil or CFI Plan Apo VC 20×/0.75 lens.

## RESULTS AND DISCUSSION

**Fig. 1:**
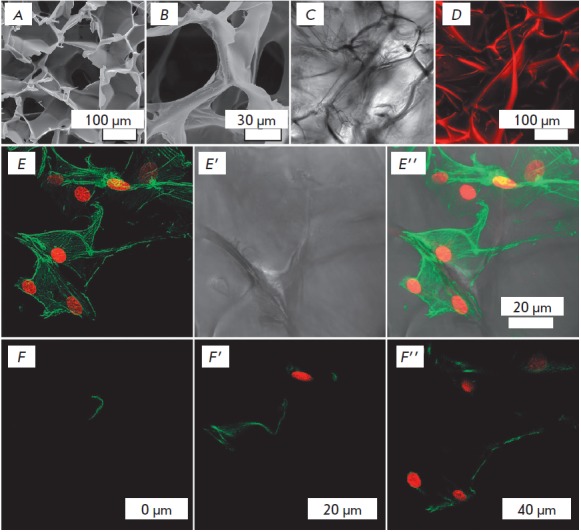
Scaffold microstructure and MEF cytoskeleton under 3D culture conditions. Scaffold images obtained by scanning electron microscopy (A–B); scaffold images obtained by transmittedlight confocal scanning in an aqueous medium (DIC, C); Z-projection of 150 optical sections of the scaffold at 1.2 μm intervals, where the scaffold material was stained with TRITC and detected using a 20x/0.75 oil immersion CFI Plan Apo VC lens (D). E–E’’ – Z-projection of 242 optical sections series at 281 nm intervals (68.002 μm) of MEF cultured on a 3D scaffold. F–F’’ – Optical sections obtained 0 (F), 20 (F’) and 40 (F’’) μm apart from the initial scanning position. The cell cytoskeleton was stained with phalloidin-FITC (green), and nuclei were stained with SYTOX orange (red). CLSM images were acquired using a Apo TIRF 60x/1.49 oil DIC objective.

**Fig. 2:**
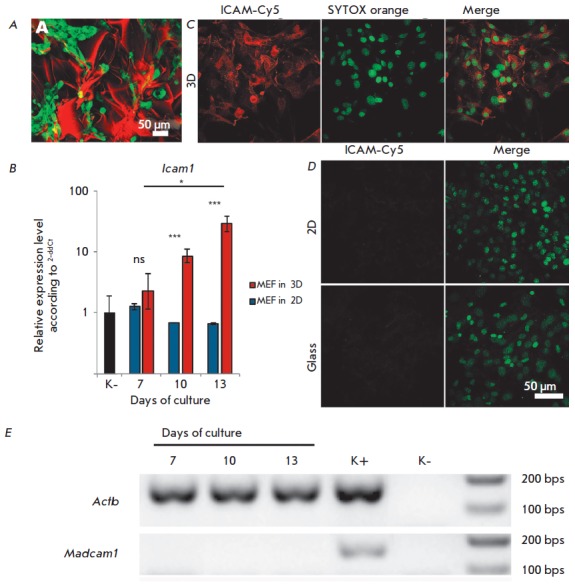
ICAM-1 expression in MEF. A – Distribution of GFP+ MEF cultured on a 3D fibroin-gelatin scaffold. The scaffold was stained with TRITC. B – Icam1 expression in MEF cultured on a 2D (plastic) and 3D fibroin-gelatin scaffold. The values were normalized on the baseline Icam1 expression level in MEF (labeled as “K-”). The data is representative of three independent experiments. * – p < 0.05; *** – p < 0.001; ns – non-significant difference. C, D – Immunofluorescence staining of ICAM-1 in MEF cultured on a 3D scaffold (C), 2D fibroin film (D, top row), and on a culture plastic surface (D, lower row). CLSM images were acquired using CFI Plan Apo VC 20x/0,75 lens. E – Expression of the Madcam1 gene in MEF cultured on a 3D fibroin-gelatin scaffold. Agarose gel electrophoresis of PCR products with specific primers to the specified genes is shown. Positive control (K +) – the material from murine lymph nodes; negative control (K-) – no cDNA was added to the PCR mix.

Fibroin gelatin scaffolds have a three-dimensional porous structure characterized by complex internal and external topographies *(Fig. 1 A–D).* Importantly, when MEFs are cultured on scaffolds, the interaction of the cell surface with the substrate occurs in various directions *(Fig. 1 D,E*). Cell distribution on the surface of a 3D scaffold is also shown in *Fig. 2A*.

Since adhesion molecules play a key role in cell-to-cell and cell-to-extracellular matrix interactions, the expression of the ICAM-1 adhesion molecule was analyzed in 3D and 2D cultures to study the effect of culture conditions on the MEF phenotype. It is known that the cytoplasmic domain of the ICAM-1 molecule interacts with the actin cytoskeleton [7], and ICAM-1 molecules clustering induces their association with actin-binding adapter proteins and binding to the F-actin cytoskeleton [8]. We hypothesized that the cytoskeleton reorganization caused by MEF cultured on 3D fibroin gelatin scaffolds can alter the ICAM-1 expression. Indeed, long-term MEF culture on fibroin gelatin scaffolds, but not on culture plastic, resulted is a significant increase in the *Icam1 *gene expression (Fig. *2B*). In full agreement with the gene expression data, bright staining of MEF with αICAM1 antibodies was observed only under 3D culture conditions *(Fig. 2B)*, while in 2D cultures on FG films or on a glass surface, almost no staining was observed *(Fig. 2D)*. The presence of a very weak signal was due to baseline *Icam1 *mRNA expression in 2D cultures *(Fig. 2B*).

Next, we analyzed the gene expression of another adhesion molecule, MAdCAM-1, in order to verify the specificity of the observed effect for ICAM-1 expression. Similarly to ICAM-1, MAdCAM-1 is expressed on stromal and endothelial cells and it is one of the key participants in the immune cell migration to lymphoid organs and barrier tissues, but it is characterized by a specific induction associated with cytokine signaling [9].

We did not detect *Madcam1 *gene expression in MEFs cultured on scaffolds, which indicates the selectivity of the effect of 3D-culture conditions on the expression of the genes that encode adhesion molecules *(Fig. 2E*).

**Fig. 3:**
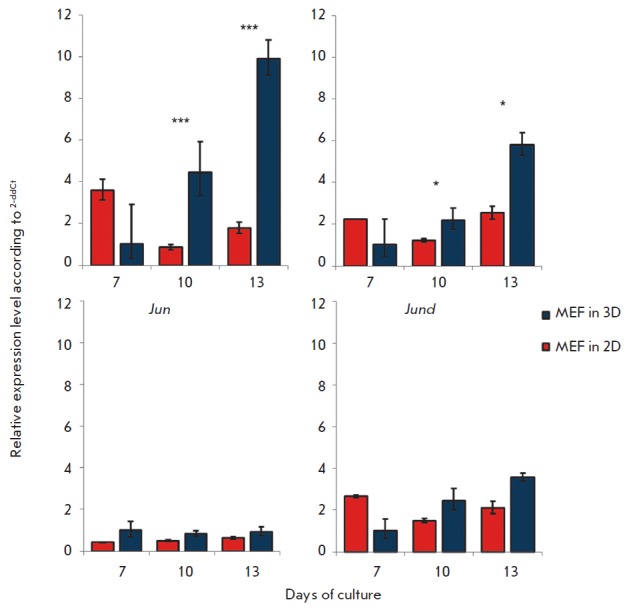
Expression of AP-1 genes. Expression of AP-1 transcription factor genes in MEF cultured on plastic (2D) and fibroingelatin scaffolds (3D). The values were normalized on the gene expression in MEF cultured under 3D conditions on day 7. * – p < 0.01; *** – p < 0.001.

It is known that the promoter region of the *Icam-1 *gene contains three binding sites for the AP-1 transcription factor, which is involved in its regulation [10]. Thus, one possible mechanism of ICAM-1 induction in MEF might be the altered activity of AP-1. The analysis of the expression of the genes that encode AP-1 subunits *(Fos, Jun, Jund, Junb) (Fig. 3)* demonstrated a significant increase in the level of *Fos *and *Junb *expression under 3D culture conditions as compared to 2D. At the same time, *Jun *and *Jund *expression did not depend on the culture conditions and did not change significantly.

Further investigations should explore which signaling pathways, starting from the mechanistic reception of 3D scaffolds by fibroblasts or intercellular interactions, could lead to AP-1 induction, followed by ICAM-1 overexpression. In addition, other transcription factors could potentially be involved in the induction of ICAM-1 overexpression in MEF. For example, it is known that NF-χB can regulate ICAM-1 expression [11].

## CONCLUSION

The culture of MEF in 3D fibroin-gelatin scaffolds leads to a significant increase in the ICAM-1 expression.

The increase in ICAM-1 expression is associated with the 3D structure of the scaffold rather than the influence of fibroin degradation products, since culturing on 2D fibroin films did not affect the expression of ICAM-1 in MEF.

Increased ICAM-1 expression is associated with increased expression of AP-1 genes, *Fos *and *Junb, *but not *Jun *and *Jund*.

## Abbreviations

2D: two-dimensional culture conditions
3D: three-dimensional culture conditions
MEF: mouse embryonic fibroblasts
FB: fibroblasts
ECM: extracellular matrix
FG: fibroin scaffold supplemented with 30% gelatin
